# Should We Strive to Make Science Bias-Free? A Philosophical Assessment of the Reproducibility Crisis

**DOI:** 10.1007/s10838-020-09548-w

**Published:** 2021-04-22

**Authors:** Robert Hudson

**Affiliations:** Department of Philosophy, University of Saskatchewan, 9 Campus Drive, Saskatoon, SK S7N 5A5 Canada

**Keywords:** Reproducibility crisis, Publication bias, Statistical significance, Value-ladenness, Heather Douglas, Kevin Elliott

## Abstract

Recently, many scientists have become concerned about an excessive number of failures to reproduce statistically significant effects. The situation has become dire enough that the situation has been named the ‘reproducibility crisis’. After reviewing the relevant literature to confirm the observation that scientists do indeed view replication as currently problematic, I explain in philosophical terms why the replication of empirical phenomena, such as statistically significant effects, is important for scientific progress. Following that explanation, I examine various diagnoses of the reproducibility crisis, and argue that for the majority of scientists the crisis is due, at least in part, to a form of publication bias. This conclusion sets the stage for an assessment of the view that evidential relations in science are inherently value-laden, a view championed by Heather Douglas and Kevin Elliott. I argue, in response to Douglas and Elliott, and as motivated by the meta-scientific resistance scientists harbour to a publication bias, that if we advocate the value-ladenness of science the result would be a deepening of the reproducibility crisis.

## Introduction

In 2016, a “News Feature” in the journal *Nature* written by Monya Baker (2016a) describes the results of a survey of 1576 scientific researchers, drawn from a wide swath of disciplines, on the topic of the reproducibility of results published in scientific literature. The survey revealed that, in the opinion of these researchers, scientific results are far less reproducible than they should be. In total, 90% of those surveyed asserted that the situation is a crisis, one that is either ‘significant’ (52%) or ‘slight’ (38%); only 3% denied the presence of a crisis (7% ‘don’t know’). A convergent, empirical observation is recorded by Daniele Fanelli who finds in a Web of Science search of the phrases ‘reproducibility crisis’, ‘scientific crisis’, ‘science in crisis’, ‘crisis in science’, ‘replication crisis’ and ‘replicability crisis’ an enormous surge of records post-2014 that ‘endorse’ the presence of a crisis (Fanelli 2018, 2629).

In general terms, philosophers are familiar with the historical fact that scientific claims, despite being initially well confirmed, often turn out to be false with continued empirical investigation. The reproducibility crisis is a fascinating instance of this phenomenon, one that has taken many contemporary scientists by surprise. Because of the crisis, many scientists from a broad spectrum of fields are faced with a methodological problem concerning the application and interpretation of statistical methods, and in so doing are finding themselves engaged with characteristically philosophical issues. What had originally been an abstract philosophical concern dealing with theory change over the course of centuries has become an unexpected, empirical problem of immediate concern.

In this context, my plan is to investigate the philosophical ramifications of the reproducibility crisis. I begin by examining the question whether there truly is a reproducibility crisis, as perhaps the issue has been overstated. After reviewing the relevant literature, and concluding that the crisis is real, I focus on the methodological issue of whether replication is in fact an ideal that should be pursued. After concluding that it is, I look at various diagnoses of the replicability crisis and conclude, along with many scientists, that a major factor in the crisis is presence of a publication bias, the fact that journals exhibit a preference for the publication of statistically significant effects as opposed to confirmations of null hypotheses, which induces scientists to seek out such effects given their need to publish for the sake of gaining academic positions, receiving promotions, winning grants, and securing professional prestige generally speaking. In response to the problem of bias, some statistical methodologists have proposed measures to counteract such bias, such as requiring journals to utilize registered reporting in which researchers submit to a journal their overall research plans for approval before collecting and analyzing data.

However, some philosophers have recently expressed the view that the goal of removing bias from science is misguided since scientific activity is fundamentally value-laden. Given the value-ladenness of science, the strategies scientists have been suggesting to ensure freedom from bias will inevitably fail. Accordingly, I review the opinions of two particular philosophers who lead the way in endorsing the value-ladenness of science, Heather Douglas and Kevin Elliott, and argue that if their views were correct, the replication crisis would be exacerbated, and the progress of science stymied. For those who reject the value-ladenness of science, such as myself, the replication crisis thus serves as an empirical touchstone that exposes an inherent flaw in the Douglas/Elliott position, one that goes beyond pure philosophical argumentation (for such argumentation, see Hudson 2016). I close the paper by considering the sort of policy initiatives Douglas and Elliott, as advocates of the value-ladenness of science, would be prone to endorse, as opposed to alternative strategies that limit the influence of bias. Considering how destructive these initiatives would be to good scientific methodology, it is clear that more measures need to be put in place to promote strategies that reduce bias and value-ladenness in science.

## Is There a Reproducibility Crisis?

In the “News Feature” cited above, Baker cites two studies that sought to replicate the results of previous experiments. In the first study, Begley and Ellis (2012) report that researchers in the company Amgen attempted to replicate 53 ‘landmark’ studies in haematology and oncology, of which they find that only 6 were successfully replicated. The second study, the Reproducibility Project headed by Brian Nosek, attempted to replicate the statistically significant effects found in 100 psychological studies published in top tier psychological journals and found that little more than one third of these attempts were successful (Open Science Collaboration 2015). There have been other replication studies since these studies, with discovered replication rates as follows (leaving aside for simplicity the issue of effect sizes): Klein et al. (2014) (Many Labs 1), 77% successful replication (sample size n = 13) in psychology; Camerer et al. (2016), 61% successful replication (sample size n = 18) in experimental economics; Ebersole et al. (2016) (Many Labs 3), 30% successful replication (n = 10) in psychology; Klein et al. (2018) (Many Labs 2), 50% successful replication (n = 28) in psychology; Camerer et al. (2018), 62% successful replication (n = 21) in social science experiments; Nosek and Errington (2017) (Reproducibility Project: Cancer Biology – early results; see Baker and Dolgin (2017), 66% (n = 3; two uninterpretable attempted replications). In summarizing the state of successful replications, Camerer et al. (2018) find an average reproducibility rate of between 35 and 75% for “published findings in social and behavioral sciences” (642), which aligns with preliminary results in cancer biology.

In their concluding appraisal of the situation, Camerer et al. (2018) see with this middling rate of replication an indication of a “systematic bias [that] is partly due to false positives and partly due to the overestimated effect sizes of true positives” (643). And generally speaking, this is the attitude scientists take towards these less than perfect replication rates, that they are a sign of a problematic feature in statistical methodology that needs to be fixed.^1^ As such, it is said that science is facing a ‘reproducibility crisis’.

However, we should point out that, for some theorists, there is not much of a crisis here at all. For example, Redish et al. (2018) argue that the failures of replication such as we have described above are a normal feature of scientific activity, and can easily occur in even tightly regimented disciplines such as mathematics and computer science, fields that are largely non-statistical. More generally, it has been argued that the history of science is itself a history of failures (Firestein 2015), and replication failures are just another instance of this phenomenon. Related to this, some take the view that replicability is overemphasized as a methodological ideal. For example, Sabrina Leonelli “[takes] issue with the widespread reference to reproducibility as an overarching epistemic value for science and a good proxy measure for the quality and reliability of research results” (2018, 131), where similar to Redish et al. she focuses on fields, such as anthropology or computer science, which are primarily non-statistical in nature. Similarly, Stephan Guttinger defends ‘localism’, according to which “it does not make sense to assume (or impose) replicability as a general epistemic criterion for the quality of scientific findings” (forthcoming, 8). Whereas scientific fields generate replicable results, others fail to do so—the matter is ‘local’ to the field at issue—and for those fields that fail to produce replicable results it is no particular cause for alarm. A scientific research area may not produce replicable results because it is too new or underdeveloped, or perhaps its subject matter is contextually sensitive in a way that investigators have not yet figured out. Thus, one might infer it is an overstatement to say that science is facing a replicability crisis in any general sense.

Nevertheless, the surprising failure to replicate many formerly well-established statistically significant results has had just such a general effect on the scientific community as it grapples with issue of what counts as an adequate empirical, statistical methodology. For instance, the American Statistical Association in 2016 provided a policy statement on “Statistical Significance and P-values”, a statement specifically motivated by the reproducibility crisis. In their preface to this policy, Executive Director to the ASA Ronald Wasserstein and Nicole Lazar comment:

The statistical community has been deeply concerned about issues of *reproducibility* and *replicability* of scientific conclusions. Without getting into definitions and distinctions of these terms, we observe that much confusion and even doubt about the validity of science is arising. (2016, 129; their italics).

Wasserstein and Lazar do not restrict the scope of their policy to any localized scientific field, nor do they counsel that the problems attending replicability are normal and to be expected. In response to the problems they cite, they recommend a more scrupulous use and interpretation of p values in setting thresholds for statistical significance. Three years later, Wasserstein, Allen Shirm and Lazar, motivated by a “perfect storm” combining “concerns about reproducible science, falling public confidence in science, and the initial impact of the ASA statement [cited above]”, provide more controversial advice, specifically, to move “beyond ‘*p* < 0.05’”, to break “free from the bonds of statistical significance” (2019, 10). This advice is endorsed in a highly cited *Nature* commentary published in 2019 with 854 scientists as co-signatories (Amrhein et al. 2019b). One benefit of this advice, according to the authors, is that “eradicating [the] categorization [between significant and non-significant claims] will help to halt... absurd statements about ‘replication failure’ when the results from the original and replication studies are highly compatible” (Amrhein et al. 2019b, 307). In other words, it is the replication crisis that motivates their proposed change: as they explain, the same experiment might generate statistically significant results at one time, and not in another, simply because of a difference in the “precision” of the studies (306). As with Wasserstein and Lazar, Amrhein et al. are not localists; they do not restrict the scope of their policy to any unique scientific field. And far from recommending that the problems attending replicability are normal and to be expected, they propose that the problems be handled by forgoing statistical significance testing altogether.

A similar recognition of a replicability crisis is found in another multiply-authored publication, this time with 72 co-authors (Benjamin et al. 2018). They start their paper stating that “the lack of reproducibility of scientific studies has caused growing concern over the credibility of claims of new discoveries based on ‘statistically significant’ findings” (2018, 6). So, once again, we see scientists from a variety of fields induced by replicability problems to propose a general change in statistical methodology. Contrary to Amrhein et al., however, their recommendation is not to dispense with statistical significance altogether but rather to impose a stricter level for α set at 0.005, instead of the usual 0.05, for new discoveries. Benjamin et al. recognize that different fields might want to impose stricter (or weaker) standards. Nevertheless, they propose for hypotheses with a prior odds of being true within a delimited range that the default α be set at *p* = 0.005 (8). This way, they suggest, the subsequent replicability of results at the *p* = 0.05 level can be improved.

There is, in fact, a third multiply-authored publication, this time with 87 co-authors, also published in 2018, that is once more a response to “the apparent non-replicability of many scientific studies” (Lakens et al. 2018, 168). Lakens and his colleagues argue that Benjamin et al.’s proposal for setting α at *p* = 0.005 may not be sufficient to improve replicability, and also has a detrimental effect on scientific practice, such as requiring larger sample sizes to meet the stricter significance level. For their part, Lakens et al. (2018) recommend either dispensing with statistical significance altogether, similar to Amrhein et al. (2019b), or alternatively proposing that scientists adopt custom-made α’s which are “determined [on a case-by-case basis] by comparing costs and benefits against a utility function using decision theory” (170). In the latter case the scientific researcher needs to justify, ahead of data collection, their choice of a particular α, which may be “higher or lower than the current convention of 0.05” (170).

The point of this discussion of the contemporary literature concerning the feasibility of statistical significance testing has been to make clear that the replicability crisis is taken seriously by many scientists, and that the failures of replication cited above are not viewed by scientists as a normal feature of scientific practice. Such failures, rather, are a problem that needs to be solved. Moreover, the policy discussions engaged in by over a thousand scientists are being developed at a general level, not locally with respect to particular subject matters. Wasserstein, Shirm and Lazar (2019), Amrhein et al. (2019b), Benjamin et al. (2018), and Lakens et al. (2018) are proposing general policies regarding how to manage what they see as an endemic problem in statistical methodology. The policy to be adopted is meant to hold across the scientific board, though it may lead to different applications in different scientific contexts.

## Why Is Replication Valued?

But then, following Leonelli and Guttinger, one might question the emphasis on replicability as an epistemic standard to begin with. To be sure, under tightly controlled conditions one might anticipate the replicability of empirical results. But one cannot expect replicability to be achievable in many scientific fields given the complexity of their subject matters and their immature states of development. Moreover, it might be the case that the best way to investigate a subject matter is not to seek replicability but rather to investigate situations where replicability fails with an eye to diagnose the reason behind this failure. From this perspective, a preoccupation with replication failure is misguided, notwithstanding the views of many scientists that a high rate of such failure constitutes a crisis.

It is worthwhile, then, to be clear about why replication, specifically, the replication of published, statistically significant effects, is important to scientific practice. In simple terms, we generally rely on the replication of observable phenomena. If I observe an object on my desk, then it is fundamental to my investigation of this object that whenever I look at the place where the object was located, under the assumption that nothing in the situation has changed, that the object is once more observed to be present. This is a simple form of replication, and it is obvious that a failure of replication in these sorts of cases would be highly disruptive to the task of learning about the object. Similar comments could be made about our investigations of any observable phenomenon. Now recall that for Camerer et al. (2018) an average reproducibility rate of between 35 and 75% for “published findings in social and behavioral sciences” would constitute a replication failure. Is this a fair statement? To put this rate in perspective, suppose that every time you checked to see whether an object is on your desk, in a case where presumably nothing has changed in any fundamental way, that you found the object to be absent one out of every four times. Clearly, such unreliable results would be highly disruptive to our attempt to learn about the object. Here I do not mean ‘unreliable’ in the sense that, in one of four times, one is not able to learn anything new about the object since it is absent. Rather, such absenteeism makes us suspicious of the reality of the object overall; normal physical objects just do not vanish every now and then. Thus, in order to be confident that there really is an object on the desk, what rate of failure would be permissible in terms of seeing that the object is there, all else being equal? It is hard to imagine that anything more than one in 1000 times would be acceptable. But we might imagine that a failure rate of at most one in 20 would serve our purposes well enough in the task of accumulating information about the world. This would correspond to an α with a p value of 0.05. And we would want to be sure that this maximum failure rate held, so we could repeat this testing a number of times.

This is analogously what is going on in cases of replication with null hypothesis significance testing. Instead of confirming whether or not there is an object on the desk, what we are trying to confirm is that there is (or is not) an appreciable correlation between two variables. This can only be established by replication, by seeing whether this correlation holds in repeated trials of an experiment. If the correlation holds, we have a stable phenomenon for investigation, and with this stability the potential to theorize about the source of the phenomenon or about how this phenomenon affects other noted phenomena. On the other hand, if the correlation fails to hold, we have an element of instability in that, for the purposes of investigation, we lack a stable phenomenon. It is as if we are investigating an object on the desk, an object that miraculously disappears every now and then. If this happens frequently enough we would suspect that the object is not physically real. So, contra Leonelli and Guttinger, replication has a clear, essential epistemic value for science and is a fundamental epistemic criterion for the quality of scientific findings. Scientific results at any level of complexity are useless, unless they are stable and predictable.

In terms of the controversy concerning whether, following Amrhein et al. (2019b), we should dispense with statistical significance altogether, or following Benjamin et al. (2018) impose a stricter level for α, or along with Lakens et al. (2018), recommend custom-made α’s established on a case-by-case basis utilizing cost-benefit analysis, the epistemic value we have just cited for replication is implicit in all these approaches. Benjamin et al.’s approach, for instance, raises the standard for when we can claim to have initially established a correlation between two variables, which improves the subsequent chance at replicability with a weaker standard. The approach of Amrhein et al. is to remove the evidential weight relating to whether an experiment can meet a statistical cut-off point, and to place instead an emphasis on alternate factors such as “background evidence, study design, data quality and understanding of underlying mechanisms [which, according to Amrhein et al.] are often more important than statistical measures such as *p* values” (2019b, 307). But then replicability of other sorts, other than a repeated ability to meet a statistical cut-off, will then come into play. Consider, for example, background evidence: such evidence needs to be counted on as having been established, and this means such evidence needs to endure despite changes of theory and the occurrence of other new pieces of evidence. That is, such evidence needs to be ‘replicable’ in terms of maintaining its epistemic status despite such changes. Similarly, with Lakens et al.’s custom-made α’s, a previously established α could become untenable with a change in the cost—benefit analysis of this α and, with that change, the epistemic status of an empirical result could become untenable as well. As such, if this result forms part of the empirical basis of a theory, this theory could be subject to question and an entire research program put in disarray—all because of a change in society’s values which alters what counts as a ‘cost’ and a ‘benefit’. So, to ensure the stability of an empirical investigation, which requires replicable results, there needs to be limits on what sort of costs and benefits could affect the setting of an α.^2^

In general, then, since the stability of results is important for scientific progress, and since such stability is assured by the practice of successfully replicating results, replication is of fundamental importance for scientific inquiry. One can then see why repeated failures of replication can be said to constitute a crisis for scientists. Scientific progress at least requires the accumulation of empirical (statistical) facts, which is difficult to achieve if such facts do not persist.

## What Is the Cause of the Reproducibility Crisis?

Why have attempted replications of previously established findings been found to fail? Here are some possible causes.

### Low π

The first thing to point out is that retrieving a statistically significant result, i.e., retrieving results with a p value less than α, should not lead one to automatically dismiss the null hypothesis since the a priori probability that the null hypothesis is false, symbolized by π, might be less than the p value. In other words, if the prior probability of an effect is low enough, what looks like a statistically significant result could well be the result of random chance, on the assumption that there is no effect and the null hypothesis is true. It follows that in subsequent attempts to replicate the experiment the effect will likely not occur as it was the unlikely product of chance to begin with. This key point is made in Bird (forthcoming) who argues that inferring an epistemic problem with statistical significance testing due to replication failures, in a case where the presence of an effect is a priori unlikely, is a form of base rate fallacy. Similar diagnoses are offered by Ioannidis (2005), Pashler and Harris (2012a) and Benjamin et al. (2018). For instance, Pashler and Harris mathematically show that, even if a statistical test has high power (e.g., 80%) and an α of 0.05, if the prior probability of the sought-after effect is only 10%, the proportion of false positive results will be 36% (2012a, 351–353). Due to the connection between the prior probability of an effect and what we can learn from a statistically significant result, Benjamin et al. (2018) recommend reducing α for new discoveries to 0.005 (with α = 0.05 for replications). With this lower α, it is less likely that the prior probability of an effect is less than α, and so less likely that a statistically significant result is purely a result of chance, i.e., a false positive.

But this diagnosis of the reproducibility crisis, and Benjamin et al.’s solution, are successful only if we have a way to determine the prior odds of an effect, or correlatively, the prior odds of the null hypothesis. Bird (forthcoming) thinks these determinations can be made if hypotheses are drawn from a field “dominated by a well-established and well-confirmed theory”, where “the hypotheses of the field test specific aspects of this theory or applications of the theory in particular domains, or they extend the theory in plausible ways” (12). Moreover, he regards particle physics as just such a field with these features, which means that its hypotheses can be expected to have high prior probabilities. (This is somewhat ironic for Bird since α is quite low in particle physics, indeed, < 0.0000003—see Bird forthcoming, 17, and also Benjamin et al. 2018, 8). By comparison, he notes that hypotheses in clinical medicine and social psychology are much more conjectural and speculative, which accounts for the higher rate of failed replications in those fields, and an increased need for a lower α. Once more, this diagnosis of the source of the reproducibility crisis only works if the identified prior probabilities are objectively accurate, not just reflections of our epistemic standpoint or purely subjective evaluations. This is because these probabilities are explaining an event that occurs in the world, such as the fact that what looks like a statistically significant effect is really a product of chance since the real presence of this effect is a priori highly unlikely. Moreover, Lakens et al. (2018) raise the further issue that determining the prior probability of an hypothesis requires specifying, to begin with, the reference class underlying this probability. They ask, is this reference class “all hypotheses in science, in a discipline or by a single researcher”? Absent an answer to this question, an estimation of the prior odds of an hypothesis has, for them, “little meaning” (169); the best we can say is that low replication rates can be explained by assuming that statistical effects have low prior probabilities, without yet a reason why we should accept the explanans in the first place.

### High α

Nevertheless, even without a prior assessment of the prior odds of an effect, one can suggest that the source of the replication crisis is utilizing an α that is too high, since the replication crisis is due to the presence of too many false positives, and the way to reduce the number of false positives is to reduce α to, say, < 0.005. But as is often noted, achieving significant results with a reduced α requires the use of much larger sample sizes, on pain of resulting in an excess of false negatives. For instance, as both Benjamin et al. (2018, 7) and Lakens et al. (2018, 169) point out, moving from α = 0.05 to α = 0.005 whilst retaining 80% power requires a 70% increase in sample size. Lakens et al. view this consequence as highly problematic in practical terms: in some areas of study there just are not enough data available due to the uniqueness of the subject matter; more generally, there are always resource limitations in terms of, at least, time and money, and so there will be fewer replications attempted and an increased use of ‘convenience’ samples, samples that are available but not necessarily the most representative of the phenomenon being studied (Lakens et al. cite the use of undergraduates and on-line samples). Benjamin et al. (2018) recognize that there is a problem here but consider the resource savings in eliminating false positives to be substantial if we thereby avoid “future studies based on false premises” (8). Bird’s response is to admit that an ultra-low α will result in the loss of some true positives for lack of an adequate sample size, but maintains that many of these missed effects will be miniscule and ultimately of no importance (forthcoming, 29).

Having a statistical test that is not sensitive enough to identify an effect, that is, one that leads to an excess of false negatives due to low power, is problematic. But there is a more serious problem that needs to be faced, one that is typically always given as, at least, a partial explanation for the replication crisis.

### Publication Bias

Modern scientific research is highly competitive. Scientists compete for jobs, funding and prestige, and the ability to acquire these things depends on one's ability to publish one’s work. Moreover, scientific journals are in competition to publish the most interesting and relevant results, and for that reason typically avoid publishing replications of previously published significant results and confirmations of null hypotheses. The result is that published scientific research exhibits a ‘publication bias’: scientists generate and report results that are publishable—i.e., statistically significant effects—and not necessarily results that are true or well-justified.

To illustrate how widespread this diagnosis of the replication crisis is, consider the influential paper by John Ioannidis with the ominous title, “Why Most Published Research Findings Are False” (2005). Ioannidis recognizes the influence of a low π and high α on replication failure. Still, he focuses heavily on the impetus to produce significant research findings as a key factor for this failure. As he defines ‘bias’, it is “the combination of various design, data, analysis, and presentation factors that tend to produce research findings when they should not be produced” (0697), but which are nevertheless produced to serve the practical ambitions of journals and scientists alike. He focuses on “the... financial and other interests and prejudices in a scientific field”, on research “conducted for no other reason than to give physicians and researchers qualifications for promotion or tenure”, and even mentions cases where “prestigious investigators... suppress via the peer review process the appearance and dissemination of findings that refute their findings, thus condemning their field to perpetuate false dogma” (0698).

These pessimistic features of modern scientific activity are concerning enough. But when combined with null hypothesis significance testing they lead to a set of practices that have a measurable negative effect on statistical inference. These practices have been highlighted by those methodologists, such as Wasserstein and Lazar (2016), Wasserstein et al. (2019) and Amrhein et al. (2019b), who recommend that significance testing be abandoned altogether. With significance testing, the goal is to collect data that meet a particular threshold, usually with α set at < 0.05, and with the pragmatic motivations cited by Ioannidis, one has various strategies one can adopt, such as “data dredging, significance chasing, significance questing, selective inference, and ‘p-hacking’ [that lead] to a spurious excess of statistically significant results in the published literature” (Wasserstein and Lazar 2016, 132; see also Wasserstein et al. 2019, 2). Publication bias has a bad influence; it is particularly pernicious when combined with “the false belief that crossing the threshold of statistical significance is enough to show that a result is ‘real’” (Amrhein et al. 2019b, 306). Benjamin et al. (2018) do not deny that there is a problem here, even with their heightened standard for α set at p < 0.005 (8). Their view, more exactly, is that the overall solution to the reproducibility crisis involves continuing with significance testing, not abandoning it, while lowering α to make it more selective.

It might be fair to say that Wasserstein et al. (2019) and Amrhein et al. (2019b) are not so much concerned with significance testing per se, but with its endemic haphazard use. Scientists in presenting their data sometimes claim to have generated significant results disproving a null hypothesis, and then confidently say they have found an ‘effect’. Alternatively, they might claim that the data are not significant, and again confidently say they have found ‘no effect’. Amrhein et al. (2019b) openly express frustration at these sorts of pronouncements. They cite 791 articles from five journals ranging across conservation biology, sociology, clinical neuropsychology and experimental psychology, half of which erroneously make such overconfident statements. As Amrhein et al. (2019b) put it, “people [should] spend less time with statistical software, and more time thinking” (307). Such a prescription is essentially the theme of Wasserstein et al. (2019) who recommend thoughtfulness in statistical analysis, such as “[considering] the scientific context and prior evidence” and “[looking] ahead to prospective outcomes in the context of theory and previous research” (4); they also encourage openness, as “providing sufficient information so that other researchers can execute meaningful alternative analyses” (6); and “being modest throughout [one’s] research, by understanding and clearly expressing the limitations of [one’s] work” (6). A publication bias leads to the violation of these prescriptions by encouraging scientists to precipitously claim to have found an ‘effect’ or ‘no effect’ on the basis of statistically significant data, without being critical enough about the quality of the data nor entirely open about how the data were acquired. In this way the replication crisis is fueled.

These sorts of concerns are common among scientists preoccupied with the quality of statistical methodology. For instance, Nosek et al. (2012) comment:

The real problem is that the incentives for publishable results can be at odds with the incentives for accurate results. This produces a conflict of interest. The conflict may increase the likelihood of design, analysis, and reporting decisions that inflate the proportion of false results in the published literature (616; see also Button et al. 2013, 365, and Smaldino and McElreath 2016, 2, for similar sentiments).

In line with this diagnosis of the crisis, there are calls to alter the incentive structure of science:

Positive, novel and clean results are more likely to be published than negative results, replications and results with loose ends; as a consequence, researchers are incentivized to produce the former, even at the cost of accuracy. These incentives ultimately increase the likelihood of false positives in the published literature. Shifting the incentives therefore offers an opportunity to increase the credibility and reproducibility of published results (Munafò et al. 2017, 7; see also Nosek et al. 2012, 617, and Ioannidis 2014, 3–5).

Alternatively, there are calls for researchers be more transparent about their collection and analysis of data. Nosek et al. (2012) call this the ‘ultimate solution”:

Three areas of scientific practice data – data, methods and tools, and workflow – are largely closed in present scientific practices. Increasing openness in each of them would substantially improve scientific progress (623; see also Button et al. 2013, 373, and Simmons et al. 2011, 1363).

To help with transparency (‘openness’ for Wasserstein et al. 2019), a common strategy is ‘registered reporting’ where researchers submit to a journal their research problem and study design for peer review before they start collecting data. As noted by the Center for Open Science, an organization co-founded by Brian Nosek and Jeffrey Spies with the mission “to increase openness, integrity, and reproducibility of research” (cos.io/about/mission/, accessed September 16, 2020), over 200 journals from a diverse set of fields, mostly from psychology and biomedicine, now include registered reporting in their submission guidelines with the aim of hindering the in-stream modification of one’s research protocol with the aim of generating a publishable result.

Pertinent to the question whether, in response to the reproducibility crisis, one should reduce α, as per the recommendation of Benjamin et al. (2018), or dispense with significance testing and α altogether, as recommended by Amrhein et al. (2019b), Ioannidis (2019a and 2019b) claims that the latter option actually increases the propensity to bias. If we assume, as with registered reporting, that the rules of research design and analysis are pre-specified ahead of time, then statistical significance can be an effective hurdle that data have to surpass to become informative. By comparison, absent a statistical threshold, it is far too easy, claims Ioannidis, for a researcher to find the presence of an effect, albeit one that is relatively small, by fitting their results into a “preexisting narrative” (2019b, 2068). Amrhein et al. respond in kind, asserting that it is in fact significance testing that “gives bias a free pass” (2019c, 1), rehearsing once more concerns about publication bias, as well as other forms of bias, such as those attending data collection which significance testing is not, in itself, equipped to deal with. Does not registered reporting serve to ameliorate the prospect of bias? Not entirely, Amrhein et al. claim: it has a ‘mitigating’ effect but, they believe, “even results from pre-registered studies can be biased by decisions invariably left open in the analysis plan” (2019b, 306). In line with the initiative to be modest about one’s findings, they also propose when dealing with single studies to dispense altogether with inferential statistics in favor of descriptive statistics (Amrhein et al. 2019c, 1).

My intent in surveying the recent methodological literature on how statistics can be modified to avoid the reproducibility crisis is simply to make the crucial point, that the problem of bias is front and center in the minds of scientists engaged with this problem. Moreover, the problem of bias is clearly epistemic: bias leads a researcher away from the truth just as bias leads to the reproducibility crisis, a situation where what scientists think is the truth in fact is not.

Two caveats. First, it is true that for some statistical methodologists the reproducibility crisis is exaggerated. A case of point is Fanelli (2018), who fails to see much bias in scientific statistical analyses, and who questions the existence of a reproducibility crisis altogether. But Fanelli's assessment, arguably, is overly optimistic. On his analysis, “p-hacking [is] common in many disciplines”, still he finds in a meta-analysis that “in the literature of all disciplines... the majority of published studies are measuring true effects” (2629), a startling claim if p-hacking is indeed common. We have seen, at any rate, that a very large number of scientists find the reproducibility crisis to be genuine and requiring a solution, presumably because it shows that something less than a majority of published studies are measuring true effects.

Secondly, Amrhein, Gelman, Greenland and McShane (2019a), in their discussion of statistical decision making in the absence of significant tests, comment that such decisions will use “explicit (even if imperfect) quantification of costs, benefits, and probabilities”. One finds a similar reference to such pragmatic, non-epistemic factors in McShane et al. (2019), where the list of ‘subordinate factors’ (as they call them), or factors that are relevant to statistical analyses, such as “prior evidence, plausibility of mechanism, study design and state data quality”, includes as well “real world costs and benefits” (235). Once more, Lakens et al. (2018) suggest, with their custom-made α strategy, to set an α level “by comparing costs and benefits against a utility function using decision theory” (170). However, in none of these papers do we find any discussion of what sorts of costs and benefits are being referred to and the degree to which such factors influence statistical thinking. Accordingly, it is unclear how we should assess the impact of these factors on the epistemic problem of bias that preoccupies so many other methodologists.

## Should We Strive to Make Science Bias-Free?

Let us say that a process of (statistical) reasoning is biased if it is influenced by factors—henceforth, ‘non-epistemic’ factors—that make the conclusion of this reasoning less likely to be true. What we have learned from the above is that scientists view bias as a problem, and strive to take steps to minimize it. In fact, there's no reason to think that, for scientists, one could not rule out bias in science altogether. At the very least, the complete removal of bias from science is an ideal that scientists strive for. If we learned that non-epistemic factors inevitably influence science, this would be very surprising and somewhat demoralizing to scientists, and to the rest of us, for it means that the processes of reasoning that occur in science are always influenced by factors that make the conclusions of this reasoning less likely to be true.

In this context, we should be attentive to an influential movement in the philosophy of science according to which science is claimed to be inevitably value-laden. The movement is expansive and contains a number of advocates, but for the sake of brevity I will review the opinions of two of its thought leaders. In her 2009 book, *Science, Policy and the Value-Free Ideal*, Heather Douglas comments that.

philosophers have... attempted to make a distinction between the kinds of values proper to scientific judgment [(i.e., epistemic values)] and the kinds of values sought to threaten the value-free ideal [(i.e., non-epistemic values)] (90).

She describes the sort of science that focuses solely on the former as ‘isolated’ science, and claims that.

the reasons for... isolated science [are] never fully articulated nor well supported. Indeed... scientific isolation from moral responsibility is unwarranted and undesirable.... It is not clear that the initial distinction between epistemic and [non-epistemic] values [is] a viable one (90).

For her, the distinction between epistemic and non-epistemic values (or factors) does not even hold up, citing the work of Rooney (1992), Longino (1996), and Lacey (1999). It follows for Douglas that “social values are influencing science through epistemic values” (2009, 91).

The same sort of view is adopted by Kevin Elliott in his 2017 book, *A Tapestry of Values*. Contrary to Douglas, Elliott allows there to be distinction between epistemic and non-epistemic values. The former concerns “things that are valued, such as accurate predictions and logical consistency” (2017, 12), whereas the latter concerns things such as “ethical or political or religious values that are typically regarded as non-epistemic” (12). For the purposes of his book, Elliott uses the term ‘values’ to cover only the latter. He then comments later in the book that

whether or not scientists consciously allow values to influence them, it is virtually inevitable that their standards of evidence will be value-laden, in the sense that they will serve some social values rather than others (2017, 99).

Focusing specifically on statistical reasoning he claims that “the best course of action is for scientific communities to acknowledge that their typical statistical approaches are value-laden” (98). As such, statistical reasoning would end up being ‘biased’, as we are using this term, since “accurate predictions and logical consistency” are not the aim of statistical decision making.

Hopefully, the views of Douglas and Elliott are now clear. As is commonly understood, non-epistemic values normally have an impact on scientific thinking. This is not denied by any of the scientists we have been examining. They are affected on a daily basis by all sorts of non-epistemic factors: social forces, moral obligations, political interests, and so on. The question is whether this impact is epistemically legitimate, that is, whether these factors can, or even should play a role in the setting and employment of evidential standards. The view of those who advocate the value-ladenness of science, such as Douglas and Elliott, is that non-epistemic values not only play this role, but should play this role. It follows that the scientists we have been examining who strive to remove the influence of bias on statistical thinking are somewhat naive in their aspirations: bias is not only inevitable in statistical analysis but should be embraced.

In terms of the evidential role non-epistemic factors play in scientific thinking, Douglas demarcates this role as either direct or indirect (2009, 96–97). The latter, indirect sort of role takes place when epistemic factors are indecisive and there is uncertainty about the extent to which they support an hypothesis. In this sort of case, Douglas argues that moral or social factors play a role in evaluating this hypothesis, filling the evidential gap. Conversely, she does not believe it is reasonable for non-epistemic values, moral or otherwise, to play a direct evidential role, where such values support an hypothesis on a “stand-alone” basis (96), independently of any antecedent epistemic support for this hypothesis. There are philosophers who do believe this, who believe that non-epistemic values do have direct evidential significance. We will not look at their views here, but only consider the more modest position Douglas is advocating.

Clearly, when dealing with statistical methodology, since the evidential support for the occurrence of a statistically significant effect is far from certain, it is legitimate on Douglas’ view for non-epistemic values to serve as indirect evidential factors, thus opening the door to moral and social values in judgments about the sufficiency of evidence on behalf of a purported effect. What impact would this have on statistical reasoning? To illustrate, here is a case of an experiment that famously failed to be replicated, from Bargh, Chen, and Burrows (1996). It involves the phenomenon of social priming, where research subjects are primed with words indicative of elderly stereotypes (i.e., they solved scrambled-sentence tasks involving words referring to commonly accepted features of old people). Subsequent to this priming, the speeds at which the research subjects leave the laboratory are surreptitiously timed, and these times are compared to analogous times exhibited by control subjects primed with words unrelated to elderly stereotypes. The surprising, statistically significant result arrived at by Bargh et al. shows that the research subjects walked out of the laboratory more slowly than the control subjects. Moreover, it is granted that Bargh et al.’s research meets established statistical standards for publication. Nevertheless, years later, Doyen, Klein, Pichon, and Cleeremans (2012) and Pashler, Coburn, and Harris (2012b) failed to replicate Bargh et al.’s result, presumably indicating that it was a false positive.

Consider, now, how Douglas would evaluate the evidential situation here. It is clearly a case where we lack certainty regarding the result, thus for Douglas it would be legitimate to import moral and social factors into our valuation of whether this is truly a case of social priming. For instance, suppose it turns out that, if occurrences of social priming are found to be statistically significant, money will become available that will provide much needed assistance for elderly walkers. On Douglas’ counsel, it would then be legitimate on evidential terms for social psychologists to adjust the level of statistical significance – that is, the risk of incurring a false positive – to ensure that social priming is statistically validated. As Douglas explains,

for any given test, the scientist must find an appropriate balance between two types of error: false positives and false negatives.... Within the parameters of available resources and methods, some choice must be made, and that choice should weigh the costs of false positives versus false negatives. Weighing those costs legitimately involves social, ethical, and cognitive values (2009, 104).

Douglas’ approach has affinities to the approach advocated by Lakens et al. (2018) with their custom-made α strategy, where α is set by reference to the ‘costs and benefits’ of making a decision. Unlike Douglas, however, Lakens et al. are not explicit that non-epistemic values have evidential relevance. For his part, Kevin Elliott is explicit about this and provides a similar assessment to the one offered by Douglas, referring to a case where scientists are performing animal studies regarding the toxicity of dioxin. He says,

if one were very concerned about promoting the economic success of the chemical industry, one could raise the statistical significance level in studies even higher than 95% so that it would be harder to declare dioxin to be harmful.... Alternatively, if one thought that public health should be valued particularly highly, one might call for lowering the statistical significance level for individual studies (2017, 95; see also 98).

Elliott clearly endorses the sort of adjustments to the level of statistical significance recommended by Douglas, where α is arbitrarily manipulated so as to promote what is perceived to be a morally or socially worthwhile cause. Contrary to Douglas, he also maintains that non-epistemic values have direct evidential significance. We leave aside this more controversial aspect of his position.

Now, is what Douglas and Elliott are proposing here controversial? Not at all, if the brunt of their assessments is simply that, for pragmatic reasons, we might be induced to vouch for the hypotheses under consideration, either that social priming is effective or that dioxin is harmful, without the further claim that these pragmatic reasons provide *evidence* for these hypotheses. But Douglas and Elliott are not saying just this. Again, for them, moral and social factors play a role in setting and employing evidential standards; they fill the evidential gap for or against an hypothesis, when the evidential situation regarding this hypothesis is uncertain. In short, pragmatic benefits can provide a reason to believe, or justify, an hypothesis. In this regard, Douglas and Elliott are taking a stand in a long debate in the history of philosophy between those who believe that pragmatic factors can justify hypotheses and those who deny this, a debate that extends back to the question of the legitimacy of Pascal's Wager as a proof of the existence of God, to the controversy between Clifford and James on what counts as evidence for the existence of God, to recent debates about the pragmatic encroachment on knowledge, and in many other places.

It is in this context that the replicability crisis can be instructive for philosophers, such as Douglas and Elliott, who subscribe to the value-laden view of science. The replicability crisis has shown that it can be extremely difficult to effectively replicate experimental results, even results that have initially met rigorous statistical standards. Of course, it has always been known that large-scale theoretical hypotheses are susceptible to disconfirmation given the further acquisition of evidence. It is now realized that even much more delimited statistical hypotheses, carefully tested and peer-reviewed, are also highly susceptible to empirical refutation. There is, as we saw, an on-going debate about the source of these failures to reproduce confirmatory evidence, and I have identified ‘publication bias’ as a widely acknowledged, tangible culprit. Still, whether or not publication bias is the main culprit behind replicability problems, it is clear that it can exacerbate such problems, as can other non-epistemic values wherever they impact the empirical evaluation of statistical hypotheses. This is because non-epistemic values have no necessary connection to the truth. It is also because their impact is variable: whether such values apply depends on the social or moral context, and this context can differ from place to place and over time. In response, many scientists feel it is necessary to introduce measures to counteract the influence of publication bias; and we should expect them to recommend similar measures wherever non-epistemic values intrude on the evidential evaluation of statistical hypotheses. But Douglas and Elliott, for their part, wholly endorse the influence of non-epistemic values in such evaluations: again, on their view, if pragmatic factors motivate one to lighten evidential standards so as to support an hypothesis, then it is legitimate to increase α to a higher p value to bring this about. Clearly such a move increases the risk of false positives since, given the variability of social and moral contexts it may, at different times, become useful or pragmatic to endorse a more restrictive α. With this variable application of standards we have a prescription for replication failure.

Yet we are currently facing a replication crisis, one which is undermining scientific progress and increasing distrust in science. We have a crisis because, without successful replications, we cannot count on the stability of empirical results, and without this stability we lack the foundation on which to construct useful theoretical hypotheses. It follows, I argue, that we should reject the value-ladenness of science as it is propounded by Douglas and Elliott, if we wish to ensure the continued replicability of statistically significant results. The importation of non-epistemic factors into the justification of hypotheses, whether in the form of a publication bias or other such factor, is an obstacle to the successful replication of statistically significant results.

## Conclusion

During the course of this paper I have argued that the replication of empirical results is a fundamental part of scientific methodology. It follows that repeated replication failures of purportedly high-quality experiments pose a significant risk for scientific research. For this reason, a number of scientists have promoted strategies aimed at countering the influence of bias on statistical methods. Two such strategies involve, first of all, making α more restrictive in terms of setting *p* < 0.005 (Benjamin et al. 2018), or alternatively, doing away with significance testing altogether in favor of a more subtle forms of statistical reasoning (Amrhein et al. 2019b).

On the other hand, Douglas and Elliott consider bias to be endemic to statistical reasoning. Where there is uncertainty in reasoning on the basis of empirical evidence, moral or social values are needed to fill the relevant ‘evidential gap’, and this is clearly the situation we are facing in statistics. I have argued that, if Douglas and Elliott are correct in their assessments, the reproducibility crisis is inevitable.

But suppose, for the sake of argument, we give Douglas and Elliott the option of setting policies to counter the reproducibility problem. What would they suggest? It is the view of Douglas and Elliot that scientists have, for far too long, ignored the moral and social implications of scientific practice. So, when it comes to a scientific issue for which there is uncertainty—and all statistical inferences are of this nature—it is the duty of scientists to adopt policies that respect our moral and social obligations. For instance, recall Elliott’s example concerning dioxin, where “if one thought that public health should be valued particularly highly, one might call for lowering the statistical significance level for individual studies” (2017, 95). Once again, this leads us to a situation where we might conclude that dioxin has harmful effects, not because the empirical evidence is particularly compelling for this conclusion, but because dioxin is a very dangerous substance. Again, the view of Douglas and Elliott is *not* that when evidence is uncertain scientists leave it to policy makers to make decisions on a pragmatic basis—that is the viewpoint of those who uphold the value-free ideal. Rather, their view is the controversial one, that the moral, social or other non-epistemic consequences of scientific decisions are themselves evidential in deciding what conclusions to draw.

Accordingly, where reproducibility problems have been identified as due to a publication bias, Douglas and Elliott’s policy recommendation would simply be to change the relevant bias at stake, preferably to one that is morally or socially meritorious. And why not? Bias is present whatever the case, since non-epistemic values are evidential, so instead of inferring statistical hypotheses that increase one’s chance of leading to a publication, infer those statistical hypotheses that lead to the best moral or social consequences. For Douglas, at least, the legitimacy of this policy depends on the uncertainty of the evidential situation—but this is a feature of statistics anyway. Conversely, for those who claim that non-epistemic factors have direct evidential impact, this policy would apply even when inferred conclusions were certain. Either way we are led to endorse polices that undermine the progress of science since, with the inevitable flux of social and moral values and the irrelevance of such values to the truth of an hypothesis under test, such policies exacerbate reproducibility problems and destabilize the empirical basis for scientific theorizing.

## Footnotes

1 As an anonymous referee correctly points out, there are other causes of replicability problems that one might cite here, beyond issues of statistical methodology. See, for example, discussions in Begley (2013), Collins and Tabak (2014), Baker (2016b) and Baker (2016c).

2 An anonymous referee correctly points out that results maybe unstable, not simply because of an instability in the methods used, but because the phenomenon of interest is unstable in itself. Thus, it is not necessarily the case that unstable phenomena are unreal, or of no scientific interest.
